# Posttraumatic Stress Reactions in Parents of Children Esophageal Atresia

**DOI:** 10.1371/journal.pone.0150760

**Published:** 2016-03-08

**Authors:** Morgane Le Gouëz, Luis Alvarez, Véronique Rousseau, Philippe Hubert, Véronique Abadie, Alexandre Lapillonne, Elsa Kermorvant-Duchemin

**Affiliations:** 1 AP-HP, Hôpital Universitaire Necker-Enfants malades, Service de Pédiatrie Générale, Paris, France; 2 Université Paris Descartes, Faculté de médecine, Paris, France; 3 AP-HP, Hôpital Universitaire Necker-Enfants malades, CPDPN, Paris, France; 4 AP-HP, Hôpital Universitaire Necker-Enfants malades, Service de Chirurgie Pédiatrique, Paris, France; 5 AP-HP, Hôpital Universitaire Necker-Enfants malades, Service de Réanimation Pédiatrique, Paris, France; 6 AP-HP, Hôpital Universitaire Necker-Enfants malades, Service de Néonatologie, Paris, France; Centre Hospitalier Universitaire Vaudois, FRANCE

## Abstract

**Objective:**

The aim of this study was to investigate psychological stress in parents of children with esophageal atresia and to explore factors associated with the development of Posttraumatic Stress disorder (PTSD).

**Design:**

Self-report questionnaires were administered to parents of children with EA. Domains included: (1) sociodemographic data, current personal difficulties, assessment scales for the quality of life and for the global health status of the child (2) French-validated versions of the *Perinatal Posttraumatic Stress disorder Questionnaire* and of the *State-Trait Anxiety Inventory*. Associations between PTSD and severity of the neonatal course, presence of severe sequelae at 2 years of age, and quality of life and global health status of children according to their parents’ perception were studied.

**Setting:**

A Tertiary care University Hospital

**Results:**

Among 64 eligible families, 54 parents of 38 children (59%) participated to the study. PTSD was present in 32 (59%) parents; mothers were more frequently affected than fathers (69 vs 46%, p = 0.03). Four mothers (8%) had severe anxiety. PTSD was neither associated with neonatal severity nor with severe sequelae at 2 years. Parents with PTSD rated their child’s quality of life and global health status significantly lower (7.5 vs 8.6; *p = 0*.*01* and 7.4 vs 8.3; *p = 0*.*02* respectively).

**Conclusions:**

PTSD is frequent in parents of children with esophageal atresia, independently of neonatal severity and presence of severe sequelae at 2 years of age. Our results highlight the need for a long-term psychological support of families.

## Introduction

Esophageal atresia (EA) is a severe congenital anomaly characterized by a lack of continuity of the esophagus; it is associated with cardiac, renal, anal or squeletal malformations in half of infants [1]. Survival of affected neonates has improved remarkably in the last decades, at the cost of important long-term respiratory, digestive, nutritional and orthopedic morbidities [2,3] including severe gastro-esophageal reflux, chronic dysphagia, asthma, tracheomalacia and scoliosis [2,3]. Little is known, however, about the psychological impact of EA, especially in parents of affected children [4].

Posttraumatic stress disorder (PTSD) is a condition following a traumatic event, where the individual presents 3 core characteristic symptoms that are intrusive thoughts and persistent symptoms of re-experiencing the traumatic event, avoidance manifestations, irritability and other symptoms of increased arousal [5]. These symptoms may impact parental attachment patterns and parent-child interactions [6,7], and subsequently the child’s development and outcomes [8]. There is evidence suggesting that parents of ill and/or injured children are at considerable risk for PTSD [9], particularly parents of premature infants [6,8,10] or of fetus diagnosed prenatally with a congenital heart disease [11,12].

Diagnosis of EA, whether it occurs at birth or prenatally, is a potentially stressful event for both mothers and fathers. Two studies have examined mothers of children with congenital anomalies including a small number of EA, reporting PTSD in 20 to 30% of mothers [13,14]. However, no studies have specifically investigated PTSD in families of children with EA.

The objectives of our work were to characterize psychological stress in parents of children with EA and to explore factors potentially associated with the development of PTSD.

## Materials and Methods

### Procedure

**Fig 1:**
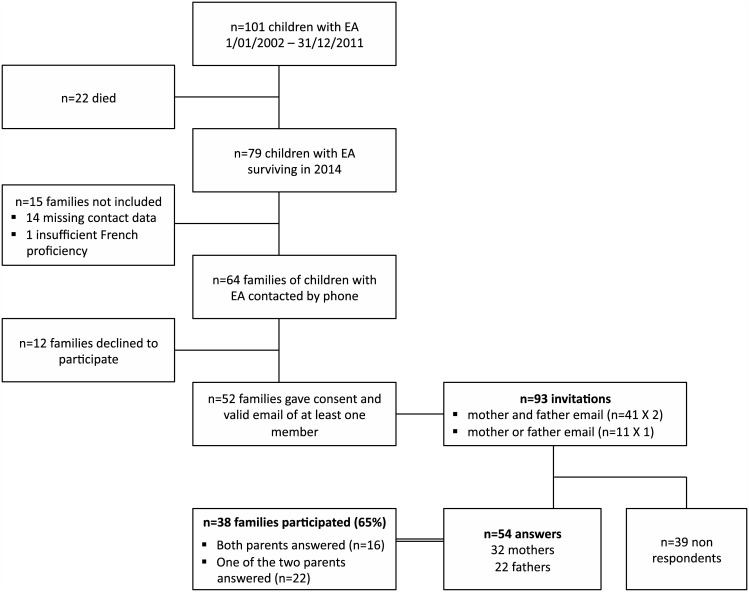
Flowchart of the participating mothers and fathers.

Eligible parents were parents of a cohort of 101 neonates with EA admitted to our tertiary center for neonatal surgical repair between January 2002 and December 2011. The flowchart in Fig 1 shows inclusion and exclusion details of eligible parents. Exclusion criteria included child’s death, insufficient French language proficiency and missing contact data. Eligible parents were contacted by phone between June and November 2014 and were given information on the aim of the study and invited to participate. Parents who gave their verbal consent to participate and provided their email address were sent by email an invitation to answer a personal self-rating questionnaire, which was made available online using the software LimeSurvey (http://www.limesurvey.org/), with an accompanying letter describing the purpose of the study. Two subsequent mailings to non-respondents were sent between August and December 2014. All responses received before February 1st, 2015 were included in the analysis.

The questionnaire consisted in three parts: (1) the first part included sociodemographic data (gender, age, marital status, educational level, work position) and actual personal difficulties (health problems, bereavement, professional or financial difficulties, couple troubles, other difficulties); (2) in the second part, participants were asked to assess the quality of life and the global health status of their child using numeric assessment scales ranging from 0 (very bad) to 10 (very good); (3) the third part included the French-validated versions of the following standardized psychometric methods: *Perinatal Posttraumatic Stress disorder Questionnaire* [5] and *State-Trait Anxiety Inventory* [15].

The French law (*Article L1121-1*, *Law n°2011–2012*, *December 29*, *2011-art*. *5*) does not require ethical approval for non-interventional research such as this study. All subjects participated voluntary; anonymity and confidentiality were maintained during the survey. This study was declared to the French National Committee on Personal Data and Privacy (*Commission Nationale de l’Informatique et des Libertés*) (number 1884521).

### Measures of parental psychological responses

#### Posttraumatic stress

The *Perinatal Posttraumatic Stress Disorder Questionnaire (PPQ)* was used to evaluate posttraumatic reactions. The PPQ is a well-validated standard personal self-rating questionnaire specially designed for parents of high-risk infants in the perinatal period. It can be administered anytime after the perinatal period [5]. The French version of the scale has been validated [5]. The PPQ is based on the *DSM-IV* diagnostic criteria and consists of 14 items, evaluating symptoms of re-experiencing and intrusive thoughts and memories, avoidance manifestations, and increased-arousal symptoms. Questions are retrospective; parents are asked to respond concerning symptoms which have appeared since birth of their child and which lasted more than 1 month. Each of the 14 items calls for a closed yes (1) or no (0) response. The posttraumatic reaction index corresponds to the sum of positive responses; a total PPQ score equal to or above 6 defines a PTSD [5].

#### Anxiety

*The State-Trait Anxiety Inventory (STAI)* has been used widely to assess anxiety symptoms with regard to both current symptoms (state STAI) and personality (trait STAI). It is a 40-item self-report scale. The answers are scored on a 4-point Likert scale. Higher scores reflect more anxiety [15]. A state or trait STAI score above 55 was used to define severe anxiety [16].

### Children data

Children data were retrospectively collected from hospital and follow-up medical charts, including perinatal data (prenatal diagnosis, gestational age, birth weight, associated malformations, medical and surgical management, duration of mechanical ventilation, duration of hospital stay) and presence of severe sequelae at 2 years of age, defined by severe delayed psychomotor development and/or severe respiratory symptoms or asthma and/or severe digestive sequelae (severe dysphagia or need for enteral nutrition) [2].

### Neonatal severity score

To reflect the seriousness of the initial perinatal course, and in the absence of a severity score specific to EA, we built a home-made non-validated score ranging from 0 to 10 based on risk factors for morbidity in EA [17]: gestational age < 34 weeks, intrauterine growth restriction, 2-step EA repair, severe cardiac malformation, ano-rectal malformation, presence of 3 or more associated abnormalities, duration of respiratory support > 7 days, length of hospital stay > 40 days, occurrence of 2 or more surgical and/or medical complications, need for enteral nutrition at discharge. Each item scored one point.

### Statistics

Continuous data were expressed as mean +/- standard deviation, and categorical variables as numbers and percentages. Bivariate analyses were performed using chi-square statistics, Fisher’s exact test or Student t test as appropriate. Linear regression analyses were performed with PTSD (PPQ score ≥ 6) as endpoint. The analyses were performed with R software. Significance was set at p<0.05.

## Results

### Participants

Among parents of the 79 alive children at the time of the study, 15 families could not be included because of missing contact details or insufficient French proficiency, and 12 declined to participate (Fig 1). A total of 93 invitations in 52 families were sent out. Fifty-four parents of 38 infants (73%) answered (Fig 1); these answers were complete and could all be used for the present study.

Characteristics of the 32 participating mothers and 22 participating fathers are summarized in Table 1. The children included in the study had a mean age of 7 (+/- 3) years at the time of survey. Their perinatal characteristics and outcomes at 2 years of age are indicated in Table 2. They were not different from the characteristics of the infants whose parents did not participate (Table 2).

**Table 1:** Baseline characteristics of the 54 participating parents.

	No. (%) or Mean (± SD)
**Parental characteristics**	
Mothers	32 (59%)
Fathers	22 (41%)
Age (years)	40 (± 5.5)
In relationship	47 (87%)
Number of children	2 (± 1)
Educational level ≥ high school diploma	51 (94%)
Executive	25 (46%)
In employment	44 (81%)
Current personal difficulties	18 (33%)

**Table 2:** Baseline characteristics of the participants’ children compared with non-participants’.

Children’s characteristics	Respondents	Non participants	*p*
Girls/Boys ratio	16/22	19/22	*0*.*9*
Age (years) at the time of the study	7 (± 3)	7.5 (±3,5)	*0*.*5*
Prenatal diagnosis	13 (34%)	18 (44%)	*0*.*5*
Associated malformations (one or more)	25 (66%)	23 (56%)	*0*.*5*
Premature birth (GA < 34 weeks)	6 (16%)	5 (12%)	*0*.*9*
Birth weight (g)	2557 (±588)	2596 (±781)	*0*.*8*
2-step surgery	4 (10.5%)	9 (21.9%)	*0*.*2*
Respiratory support duration ≤ 7 days	22 (58%)	19 (46%)	*0*.*3*
Length of stay ≤ 40 days	19 (50%)	19 (46%)	*0*.*9*
Artificial enteral nutrition at discharge	11 (29%)	11 (27%)	*1*
Neonatal severity score (/10)	2.4 (± 2)	2.6 (±2)	*0*.*6*
Severe sequelae at 2 years *	9 (25%)	12 (35%)	*0*.*5*

* Children lost for follow-up: 2 in participants, 7 in non participants.

### Parents’ psychological responses

The mean State-STAI score was 48 (± 5) (range 36 to 64) with no difference between mothers and fathers. Four mothers (7.5%) had a State-STAI score above 55, reflecting a severe state anxiety (Table 3). One mother also had a Trait-STAI above 55, reflecting an anxiety-related personality disorder.

**Table 3:** Parental psychological responses.

	Mothers (n = 32)	Fathers (n = 22)	*p*
**State-STAI score**, mean (± SD) (range)	49 (± 5.7) (36–64)	47 (± 4.4) (41–54)	*0*.*2*
**State-STAI > 55**, n (%)	4 (12.5%)	0	*0*.*1*
**Trait-STAI score**, mean (± SD) (range)	46 (± 5.3) (38–62)	46 (± 4.8) (34–55)	*0*.*8*
**Trait-STAI > 55**, n (%)	1 (3%)	0	*1*
**PPQ score**, mean (± SD) (range)	7 (± 3.7) (0–13)	4,7 (± 3.5) (0–10)	*0*.*03*
**PPQ ≥ 6**, n (%)	22 (69%)	10 (45.5%)	*0*.*03*

Thirty-two parents (59%) had a PPQ score equal to or above 6, i.e. in the clinical range of PTSD. Twenty five (65%) child—parent pairs contained at least one member who scored above cut-off. As shown in Table 3, a significantly higher proportion of mothers (n = 22, 69%) than fathers (n = 10, 46%) had clinical PTDS symptoms (*p = 0*.*03*). No significant differences in parental psychological responses were found depending on socio-demographic characteristics and perinatal characteristics of the children, including prenatal diagnosis (Table 4). In particular, presence of PTSD symptoms was neither correlated with the neonatal severity score (*R*^*2*^ *= 0*.*002*, *p = 0*.*76)* nor with the presence of severe sequelae at 2 years of age in the infant (Table 4). Though not significant, mothers of children with lower (below 5/10) neonatal severity scores tended to have higher PPQ score (7.3 vs 5.8/14 compared to mothers of children with neonatal severity scores > 5, *p = 0*.*4*). PTDS was weakly associated to the age of the child at the time of survey (*R*^*2*^ *= 0*.*07*, *p = 0*.*046*), tending to decrease with time (Table 5). Parents with PTSD rated significantly lower scores of quality of life and global health status scales in their child (7.5 vs 8.6; *p = 0*.*01* and 7.4 vs 8.3; *p = 0*.*02*, respectively). Linear regression analysis showed that parents’ perception of the quality of life and of the global health status of their child was significantly associated with PTSD (Table 5). No association was found between PTSD and anxiety (state or trait) scores (Table 5).

**Table 4:** Distribution of independent variables per PTSD status.

	PTSD	no PTSD	*p*
**Parental characteristics**	n = 32 parents	n = 22 parents	
Unemployed	1	2	*0*.*6*
Low educational level	6	5	*0*.*7*
Current personal difficulties	14	4	*0*.*09*
**Children characteristics**	n = 25 children	n = 13 children	
Age of child at the time of survey (years), mean (±SD)	6.8 (±3)	7.3 (± 4)	*0*.*7*
Prenatal diagnosis, n (%)	9 (36%)	4 (31%)	*1*
2-step surgery	3 (12%)	1 (7.7%)	*1*
Associated malformations (one or more), n (%)	17 (68%)	8 (62%)	*0*.*7*
Premature birth (GA < 34 weeks), n (%)	2 (8%)	4 (30%)	*0*.*2*
Respiratory support duration ≤ 7 days, n (%)	16 (64%)	6 (46%)	*0*.*5*
Length of stay ≤ 40 days, n (%)	12 (48%)	7 (54%)	*1*
Neonatal severity score (/10), mean (±SD)	2.3 (± 1.8)	2.6 (± 2.6)	*0*.*7*
Severe sequelae at 2 years, n (%)	6 (25%)	3 (25%)	*1*

**Table 5:** Correlation between (A) parents’ PPQ score and parents’ STAI (anxiety) scores and correlation (B) between parents’ PPQ score and quality of life and global health status in children as reported by their parents, and the child’s age at the time of survey.

Variable	Coefficient (R^2^)	*p*
Trait-STAI	0.00007	*1*
State-STAI	0.01	*0.4*
Quality of child’s life scale	0.21	*0.0004*
Child’s global health status scale	0.18	*0.001*
Child’s age at the time of survey	0.07	*0.046*

## Discussion

This is the first study to examine specifically PTSD and anxiety in parents, including both mothers and fathers, of surviving children with EA. Our results suggests that post-traumatic stress disorder is a very frequent reaction in parents of newborns with EA, affecting both mothers and fathers. Overall, 2/3 of child—parent pairs contained at least one member who scored above cut-off; 59% of all participants did. In contrast, severe state anxiety was observed less frequently, affecting 4 (7.5%) parents, although the mean STAI score for state anxiety was high among participants (48 ±5). Indeed, parents of neonates born with an EA face stressful and emotionally demanding experiences over time [4], including diagnosis of a congenital anomaly and frequent early separation since the baby’s referral to another medical center is often necessary due to high rates of postnatal diagnosis. Newborns with EA most often undergo surgery within 24 hours of birth; they all require neonatal intensive care and sometimes need prolonged hospitalization. Although in different settings and using various scales to score PTSD, previous studies examining psychological distress in parents of sick or injured children have shown similar high levels of PTSD. In parents whose child was admitted in a PICU, Nelson and al. [18] report a prevalence rate of PTSD between 10 and 21%, with subclinical PTSD as high as 84%. Nearly 30% to 40% of mothers of children with cancers [19,20] and almost half of the mothers after a very preterm delivery [6–8] experience PTSD symptoms.

One of the strengths of our work is that it included both mothers and fathers while most other studies on psychological distress focused on the reactions of mothers only. Mothers were more exposed to PTSD than fathers, consistently with previous data in parents of premature infants [8], acutely ill children [18,20] or with a congenital anomaly [14]. This difference in the pattern of the trauma response may be due to the mother’s stronger perceptions of threat and loss of control and to a gender-specific psychobiological reaction to trauma [21,22]. Mothers may also be more exposed to a feeling of guilt that is often reported when a malformation or a disease is diagnosed in their child, which could potentiate posttraumatic stress disorders [23]. It has also been reported that fathers tended to minimize their infant’s medical problems and used instrumental coping strategies to reduce stress [24].

An unexpected and striking finding was that presence of PTSD symptoms was neither associated with the severity of the neonatal course nor with presence of severe sequelae at 2 years of age, with some parents developing PTSD while their child had an uneventful course. Although a lack of power to show a difference cannot be excluded, the raw data did not support this hypothesis, showing lower neonatal severity scores in children of mothers with PTSD and the same rate of severe sequelae in both groups. Several published studies in acutely ill children have yielded similar results regarding the relationship between illness-related variables and subsequent parent psychological distress [9,18,25]. Notably, Balluffi et al. found, in a prospective study in parents of children admitted to a PICU, that PTSD was not associated with objective measures of the infant illness severity assessed by the Pediatric Risk of Mortality score [9]. In premature infants, a number of studies have shown there was no relationship between PTSD in parents and the degree of neonatal illness, assessed by Perinatal Risk Inventory score [5,8] or the Score for Neonatal Acute Physiology [26]. It has been argued that distress may be more related to subjective than objective factors, such as parental perception of their infant’s illness severity [9,18,27]. An hypothesis to explain these observations may be that parents of babies with uneventful perinatal course may not have received as much attention and support from staff during their child’s hospitalization as parents of sicker infants. While we could not assess parental views on the overall quality of medical-surgical and nursing care nor the quality of caregivers support during the initial hospital course of their child reliably, future research could test the relevance of this association, in a perspective of PTSD prevention during the NICU stay.

In our study, PTSD symptoms were found several years after the infant’s birth. Research on long-term parental psychological responses is scarce, in particular in relation to congenital anomalies, but data suggest that clinically important psychological distress persists several years after the traumatic event. In a study assessing long-term psychological responses among parents of children with a congenital anomaly, 30% of parents (36% of the mothers and 23% of the fathers) still reported clinically important psychological distress 9 years after birth [14].

High PTSD scores were significantly associated with a worse subjective evaluation by the parents of their child’s global health status and quality of life, suggesting that PTSD influences parental perception of their child’s health. Balluffi et al. also found an association between PTSD with the parent’s degree of worry that the child might die, while PTSD was independent of objective measures of the mortality risk in the child [9].

A limitation of the study is a relatively high rate of attrition, although at least one member of around 60% of the 64 families who were contacted agreed to participate. This is a frequent issue in many studies of PTSD, and is particularly relevant, since, for non-respondents, avoiding participation may indicate a worse psychological state [28]. Despite the fact that non-participants’ children had similar perinatal characteristics, age at the time of the study and rate of severe sequelae at 2 years old compared to children of participants, the assumption that PTSD and anxiety may be overvalued could also be made. Assuming no posttraumatic stress in non-participants, the PTSD rate in all eligible parents would still be as high as 20%.

We chose to limit our study to two aspects of parental psychological responses (i.e. PTSD and anxiety) to avoid a too long questionnaire and incomplete responses. Therefore, depression and feelings related to acute stress response such as guilt and anger were not evaluated. Repeated clinical interviews focusing on subjective emotional experience could provide more precise data on the quality and duration of parental stressful experience.

A number of earlier investigations in premature infants have documented that maternal psychological distress negatively affects the quality of the parent–child interactions [6,7] and possibly the child’s outcomes such as sleeping and eating behavior and development, although the perinatal risk factors do not [8]. Although the impact of parental posttraumatic stress on outcomes of children with EA remains to be investigated, our results highlight the need for a long-term psychological follow-up of families to detect parents with PTSD, in order to plan parent-focused supportive interventions in the neonatal period and beyond. In premature infants, previous studies have suggested that intervention programs, including structured psychological intervention [29], trauma-focused cognitive behavior therapy [30] or family-centered care [31] may reduce parental symptoms of trauma and psychological distress. Such interventions have not been evaluated in parents of children with EA and represent an important avenue of research, in EA but also in other congenital anomalies.

## Supporting Information

S1 File Parental questionnaire (in French). (PDF)

S2 File English version of the parental questionnaire. (PDF)
