# Supplementary material for: Posttraumatic Stress Reactions in Parents of Children Esophageal Atresia
Source: PLoS One. 2016 Mar 8;11(3):e0150760. doi: 10.1371/journal.pone.0150760 (PMC4783023; doi:10.1371/journal.pone.0150760)
Supplement: S1 File — (PDF) [file pone.0150760.s001.pdf]

# Retentissement psychologique chez les parents d'enfants opérés d'une atrésie de l'oesophage

Chers parents,

Votre enfant a été opéré d'une atrésie de l'œsophage en période néonatale à l'hôpital Necker. La prise en charge de votre nouveau-né (procédures médicales et chirurgicales, hospitalisation(s) prolongée(s), incertitudes sur le devenir de votre enfant) dès les premiers jours de sa vie a *potentiellement* été un événement traumatisant.

Mon travail de thèse de médecine a porté sur le suivi et l'évolution des enfants opérés d'une atrésie de l'oesophage. Désormais, dans le cadre de mon mémoire de Diplôme d'Etudes Spécialisées en Néonatalogie, je souhaiterais évaluer le stress parental engendré à long terme par l'hospitalisation d'un enfant en période néonatale pour atrésie de l'œsophage.

Les résultats de ce travail permettront d'adapter la prise en charge des familles en néonatologie et d'améliorer le soutien aux parents.

Ce questionnaire est constitué de 4 parties et peut être complété en 15 à 20 minutes. Votre participation est bien sûr libre et vos réponses sont anonymisées.

Je vous remercie vivement de votre participation et reste à votre disposition pour plus d'informations si vous le souhaitez.

Dr Morgane Le Gouëz

morgane.legouez@gmail.com

Dr Elsa Kermorvant

Service de Pédiatrie et Réanimation néonatales

Hôpital Universitaire Necker-Enfants malades

Il y a 65 questions dans ce questionnaire

## Informations générales

### []Vous êtes :

Veuillez sélectionner une seule des propositions suivantes :

- ○ La mère d'un enfant opéré d'une atrésie de l'oesophage
- ○ Le père d'un enfant opéré d'une atrésie de l'oesophage

### []Quel est votre âge (années) ?

Seuls des nombres peuvent être entrés dans ce champ.

Veuillez écrire votre réponse ici :

## []Quelle est votre situation familiale ?

Veuillez sélectionner une seule des propositions suivantes :

- En couple/mariés
- Séparés/divorcés
- Veuf/veuve

## []Combien avez vous d’enfants ?

Veuillez sélectionner une seule des propositions suivantes :

- 1
- 2
- 3
- 4
- 5

## []Quelle a été votre scolarité ?

Veuillez sélectionner une seule des propositions suivantes :

- Collège
- Lycée/baccalauréat
- Bac +2 ou 3
- Bac + 4 ou 5
- > bac +5

## []Quelle est votre profession ?

Veuillez sélectionner une seule des propositions suivantes :

- Agriculteur exploitant
- Artisan, commerçant, ou chef d’entreprise
- Cadre ou profession intellectuelle supérieure
- Profession intermédiaire
- Employé
- Ouvrier

## []Quelle est votre activité actuelle ?

Veuillez sélectionner une seule des propositions suivantes :

- ○ Actif
- ○ Au foyer
- ○ En congé parental
- ○ En recherche d'emploi
- ○ Elève, étudiant, ou en formation
- ○ Retraité

## []Traversez vous actuellement des difficultés personnelles ?

Veuillez choisir toutes les réponses qui conviennent :

- ☐ Aucune difficulté
- ☐ Problèmes de santé
- ☐ Maladie ou deuil d'un proche
- ☐ Difficultés d'ordre professionnel (chômage, licenciement…)
- ☐ Difficultés conjugales
- ☐ Difficultés financières
- ☐ Autres

## []Bénéficiez-vous actuellement d'un suivi psychologique ?

Veuillez sélectionner une seule des propositions suivantes :

- ○ Oui
- ○ Non

**[]Actuellement, sur une échelle de 0 (très mauvais) à 10 (très bon), comment évaluez-vous l'état de santé général de votre enfant ?**

Veuillez sélectionner une seule des propositions suivantes :

- ○ 1
- ○ 2
- ○ 3
- ○ 4
- ○ 5
- ○ 6
- ○ 7
- ○ 8
- ○ 9
- ○ 10

**[]Actuellement, sur une échelle de 0 (très mauvaise) à 10 (très bonne), comment évaluez-vous la qualité de vie de votre enfant (activités quotidiennes, loisirs, intégration scolaire, groupe d'amis...) ?**

Veuillez sélectionner une seule des propositions suivantes :

- ○ 1
- ○ 2
- ○ 3
- ○ 4
- ○ 5
- ○ 6
- ○ 7
- ○ 8
- ○ 9
- ○ 10

# PPSQ

Répondez « oui » si vous avez traversé l'une de ces expériences durant les 6 mois ayant suivi la naissance de votre bébé.

Ne répondez « oui » que si l'expérience en question a duré plus d'un mois.

**[]Avez-vous eu plusieurs fois des mauvais rêves au sujet de votre accouchement ou du séjour de votre bébé à l'hôpital ?**

Veuillez sélectionner une seule des propositions suivantes :

- Oui
- Non

**[]Avez-vous eu à plusieurs reprises des mauvais souvenirs concernant votre accouchement ou le séjour de votre bébé à l'hôpital ?**

Veuillez sélectionner une seule des propositions suivantes :

- Oui
- Non

**[]Avez-vous eu parfois soudainement l'impression de revivre la naissance de votre bébé ?**

Veuillez sélectionner une seule des propositions suivantes :

- Oui
- Non

**[]Avez-vous essayé d'éviter de penser à votre accouchement ou au séjour de votre bébé à l'hôpital ?**

Veuillez sélectionner une seule des propositions suivantes :

- Oui
- Non

**[]Avez-vous évité de faire certaines choses qui pourraient faire resurgir des émotions concernant l'accouchement ou le séjour de votre bébé à l'hôpital (comme, par exemple, ne pas regarder un programme de télévision sur les bébés) ?**

Veuillez sélectionner une seule des propositions suivantes :

- ◯ Oui
- ◯ Non

**[]Etiez-vous incapable de vous souvenir de certains moments concernant le séjour de votre bébé à l'hôpital ?**

Veuillez sélectionner une seule des propositions suivantes :

- ◯ Oui
- ◯ Non

**[]Avez-vous perdu de l'intérêt pour vos occupations habituelles (par exemple pour votre travail, votre famille) ?**

Veuillez sélectionner une seule des propositions suivantes :

- ◯ Oui
- ◯ Non

**[]Vous êtes vous senti(e) seul(e) ou à l'écart des autres (par exemple, pensiez-vous que personne ne vous comprenait) ?**

Veuillez sélectionner une seule des propositions suivantes :

- ◯ Oui
- ◯ Non

**[]Est-ce qu'il est devenu plus difficile pour vous de ressentir de la tendresse ou de l'amour avec les autres ?**

Veuillez sélectionner une seule des propositions suivantes :

- ◯ Oui
- ◯ Non

**[]Avez-vous eu une difficulté inhabituelle à vous endormir ou à rester endormi(e) ?**

Veuillez sélectionner une seule des propositions suivantes :

- Oui
- Non

**[]Etiez-vous plus irritable et colérique avec les autres que d'ordinaire ?**

Veuillez sélectionner une seule des propositions suivantes :

- Oui
- Non

**[]Avez-vous eu plus de difficultés à vous concentrer qu'avant votre accouchement ?**

Veuillez sélectionner une seule des propositions suivantes :

- Oui
- Non

**[]Etes-vous devenu(e) plus à fleur de peau (par exemple, vous êtes-vous senti(e) plus sensible aux bruits ou sursautiez-vous plus facilement) ?**

Veuillez sélectionner une seule des propositions suivantes :

- Oui
- Non

**[]Vous est-il arrivé de ressentir de la culpabilité à propos de la naissance de votre enfant sans pouvoir vous raisonner ?**

Veuillez sélectionner une seule des propositions suivantes :

- Oui
- Non

# STAI-1

Vous trouverez ci-dessous un certain nombre d'énoncés que les gens ont déjà utilisés pour se décrire. Lisez chaque énoncé, puis en sélectionnant le mot approprié sous l'énoncé indiquez comment vous vous sentez **maintenant**, c'est à dire **à ce moment précis**. Il n'y a pas de bonnes ou de mauvaises réponses.

Ne vous attardez pas trop longtemps sur un énoncé ou l'autre mais donnez la réponse qui vous semble décrire le mieux les sentiments que vous éprouvez **présentement**.

## []Présentement, je me sens calme.....

Veuillez sélectionner une seule des propositions suivantes :

- ○ Beaucoup
- ○ Modérément
- ○ Un peu
- ○ Pas du tout

## []Présentement, je me sens en sécurité.....

Veuillez sélectionner une seule des propositions suivantes :

- ○ Beaucoup
- ○ Modérément
- ○ Un peu
- ○ Pas du tout

## []Présentement, je suis tendu(e).....

Veuillez sélectionner une seule des propositions suivantes :

- ○ Beaucoup
- ○ Modérément
- ○ Un peu
- ○ Pas du tout

## []Présentement, je me sens surmené(e).....

Veuillez sélectionner une seule des propositions suivantes :

- Beaucoup
- Modérément
- Un peu
- Pas du tout

## []Présentement, je me sens tranquille.....

Veuillez sélectionner une seule des propositions suivantes :

- Beaucoup
- Modérément
- Un peu
- Pas du tout

## []Présentement, je me sens bouleversé(e).....

Veuillez sélectionner une seule des propositions suivantes :

- Beaucoup
- Modérément
- Un peu
- Pas du tout

## []Présentement, je suis préoccupé(e) par des malheurs possibles.....

Veuillez sélectionner une seule des propositions suivantes :

- Beaucoup
- Modérément
- Un peu
- Pas du tout

### []Présentement, je me sens comblé(e) .....

Veuillez sélectionner une seule des propositions suivantes :

- Beaucoup
- Modérément
- Un peu
- Pas du tout

### []Présentement, je me sens effrayé(e) .....

Veuillez sélectionner une seule des propositions suivantes :

- Beaucoup
- Modérément
- Un peu
- Pas du tout

### []Présentement, je me sens à l'aise .....

Veuillez sélectionner une seule des propositions suivantes :

- Beaucoup
- Modérément
- Un peu
- Pas du tout

### []Présentement, je me sens sûr(e) de moi .....

Veuillez sélectionner une seule des propositions suivantes :

- Beaucoup
- Modérément
- Un peu
- Pas du tout

**[]Présentement, je me sens nerveux(se) .....**

Veuillez sélectionner une seule des propositions suivantes :

- Beaucoup
- Modérément
- Un peu
- Pas du tout

**[]Présentement, je suis affolé(e) .....**

Veuillez sélectionner une seule des propositions suivantes :

- Beaucoup
- Modérément
- Un peu
- Pas du tout

**[]Présentement, je me sens indécis(e) .....**

Veuillez sélectionner une seule des propositions suivantes :

- Beaucoup
- Modérément
- Un peu
- Pas du tout

**[]Présentement, je suis détendu(e) .....**

Veuillez sélectionner une seule des propositions suivantes :

- Beaucoup
- Modérément
- Un peu
- Pas du tout

### []Présentement, je me sens satisfait(e) .....

Veuillez sélectionner une seule des propositions suivantes :

- Beaucoup
- Modérément
- Un peu
- Pas du tout

### []Présentement, je suis préoccupé(e) .....

Veuillez sélectionner une seule des propositions suivantes :

- Beaucoup
- Modérément
- Un peu
- Pas du tout

### []Présentement, je me sens tout mêlé(e) .....

Veuillez sélectionner une seule des propositions suivantes :

- Beaucoup
- Modérément
- Un peu
- Pas du tout

### []Présentement, je sens que j'ai les nerfs solides.....

Veuillez sélectionner une seule des propositions suivantes :

- Beaucoup
- Modérément
- Un peu
- Pas du tout

## []Présentement, je me sens bien .....

Veuillez sélectionner une seule des propositions suivantes :

- ○ Beaucoup
- ○ Modérément
- ○ Un peu
- ○ Pas du tout

# STAI-2

Vous trouverez ci-dessous un certain nombre d'énoncés qui ont déjà été utilisés par les gens pour se décrire. Lisez chaque énoncé, puis en sélectionnant le mot approprié sous l'énoncé, indiquez comment vous vous sentez **en général**. Il n'y a pas de bonnes ou de mauvaises réponses. Ne vous attardez pas trop longtemps sur un énoncé ou l'autre mais donnez la réponse qui vous semble décrire le mieux les sentiments que vous éprouvez **en général**.

### []En général, je me sens bien .....

Veuillez sélectionner une seule des propositions suivantes :

- Presque toujours
- Souvent
- Quelquefois
- Presque jamais

### []En général, je me sens nerveux(se) et agité(e) ....

Veuillez sélectionner une seule des propositions suivantes :

- Presque toujours
- Souvent
- Quelquefois
- Presque jamais

### []En général, je me sens content(e) de moi-même .....

Veuillez sélectionner une seule des propositions suivantes :

- Presque toujours
- Souvent
- Quelquefois
- Presque jamais

## []En général, je voudrais être aussi heureux(se) que les autres semblent l'être.....

Veuillez sélectionner une seule des propositions suivantes :

- Presque toujours
- Souvent
- Quelquefois
- Presque jamais

## []En général, j'ai l'impression d'être un(e) raté(e) .....

Veuillez sélectionner une seule des propositions suivantes :

- Presque toujours
- Souvent
- Quelquefois
- Presque jamais

## []En général, je me sens reposé(e) .....

Veuillez sélectionner une seule des propositions suivantes :

- Presque toujours
- Souvent
- Quelquefois
- Presque jamais

## []En général, je suis d'un grand calme .....

Veuillez sélectionner une seule des propositions suivantes :

- Presque toujours
- Souvent
- Quelquefois
- Presque jamais

**[]En général, je sens que les difficultés s'accumulent au point où je n'arrive pas à les surmonter .....**

Veuillez sélectionner une seule des propositions suivantes :

- Presque toujours
- Souvent
- Quelquefois
- Presque jamais

**[]Je m'en fais trop pour des choses qui n'en valent pas vraiment la peine.....**

Veuillez sélectionner une seule des propositions suivantes :

- Presque toujours
- Souvent
- Quelquefois
- Presque jamais

**[]En général, je suis heureux(se) .....**

Veuillez sélectionner une seule des propositions suivantes :

- Presque toujours
- Souvent
- Quelquefois
- Presque jamais

**[]En général, j'ai des pensées troublantes .....**

Veuillez sélectionner une seule des propositions suivantes :

- Presque toujours
- Souvent
- Quelquefois
- Presque jamais

### []En général, je manque de confiance en moi .....

Veuillez sélectionner une seule des propositions suivantes :

- Presque toujours
- Souvent
- Quelquefois
- Presque jamais

### []En général, je me sens en sécurité .....

Veuillez sélectionner une seule des propositions suivantes :

- Presque toujours
- Souvent
- Quelquefois
- Presque jamais

### []En général, prendre des décisions m’est facile .....

Veuillez sélectionner une seule des propositions suivantes :

- Presque toujours
- Souvent
- Quelquefois
- Presque jamais

### []En général, je sens que je ne suis pas à la hauteur de la situation .....

Veuillez sélectionner une seule des propositions suivantes :

- Presque toujours
- Souvent
- Quelquefois
- Presque jamais

### []En général, je suis satisfait(e) .....

Veuillez sélectionner une seule des propositions suivantes :

- Presque toujours
- Souvent
- Quelquefois
- Presque jamais

### []En général, des idées sans importance me passent par la tête et me tracassent .....

Veuillez sélectionner une seule des propositions suivantes :

- Presque toujours
- Souvent
- Quelquefois
- Presque jamais

### []En général, je prends les désappointements tellement à cœur que je n’arrive pas à les chasser de mon esprit .....

Veuillez sélectionner une seule des propositions suivantes :

- Presque toujours
- Souvent
- Quelquefois
- Presque jamais

### []En général, je suis une personne qui a les nerfs solides .....

Veuillez sélectionner une seule des propositions suivantes :

- Presque toujours
- Souvent
- Quelquefois
- Presque jamais

**[]En général, je deviens tendu(e) ou bouleversé(e) quand je songe à mes préoccupations et à mes intérêts récents ....**

Veuillez sélectionner une seule des propositions suivantes :

- ○ Presque toujours
- ○ Souvent
- ○ Quelquefois
- ○ Presque jamais

Nous vous remercions vivement de votre participation !

Envoyer votre questionnaire.
Merci d'avoir complété ce questionnaire.
