# Supplementary material for: Posttraumatic Stress Reactions in Parents of Children Esophageal Atresia
Source: PLoS One. 2016 Mar 8;11(3):e0150760. doi: 10.1371/journal.pone.0150760 (PMC4783023; doi:10.1371/journal.pone.0150760)
Supplement: S2 File — (PDF) [file pone.0150760.s002.pdf]

Supporting Information file – Le Gouez et al - English copy of the questionnaire

Cover letter

Dear parents,

Your child had surgery for esophageal atresia at Necker Hospital in his/her newborn period. The related management (medical and surgical procedures, prolonged hospitalization) and uncertainty about the future of your child may have been a stressful event for you.

My M.D. thesis work focused on the long-term outcome of children with esophageal atresia. As part of my thesis for a Specialty Diploma in Neonatal Medicine, I am now conducting a research study to learn more about the experience of parents who have had a baby hospitalized in the NICU for esophageal atresia.

The results of this work will help us provide the best support to other parents and improve family care and management.

This survey consists of 4 parts and should take you approximately 20 minutes to complete. Your participation is voluntary and you may quit the survey at any times. All of the survey responses are anonymous.

I thank you for taking the time to complete this survey and remain at your disposal for any further information.

Dr Morgane Le Gouëz

morgane.legouez@gmail.com

Dr Elsa Kermorvant

Neonatal Intensive Care Unit

Necker-Enfants malades University Hospital

Part 1 - General information

Are you:

❑ The father of a child with esophageal atresia

❑ The mother of a child with esophageal atresia

How old are you? (years)

Please fill in the box

What is your marital status? Please select one of the following proposals:

❑ Married/In relationship

❑ Divorced

❑ Widowed

How many children do you have?

❑ 1

❑ 2

❑ 3

❑ 4

❑ 5

What is the highest level of education you have completed? Please select one of the following proposals:

❑ Less than high school

❑ High school

❑ Baccalaureate degree

❑ Masters degree

❑ Doctoral or Professional degree

What is your occupation? Please select one of the following proposals:

❑ Farmer

❑ Craftsman, shopkeeper or business owner

❑ Executive or intellectual profession

❑ Intermediate occupation

❑ Employee

❑ Worker

What is your current activity? Please select one of the following proposals:

❑ Active

❑ Homemaker

❑ On parental leave

❑ Looking for employment

❑ Pupil, student, or in training

❑ Retired

Are going through personal difficulties? Please select all that apply:

❑ No trouble

❑ Health problems

❑ Illness or bereavement of a close

❑ Professional difficulties (unemployment, dismissal...)

❑ Marital difficulties

❑ Financial difficulties

❑ Other

Do you currently benefit from psychological counseling? Please select one of the following proposals:

❑ Yes

❑ No

Part 2

On a scale of 0 (very poor) to 10 (excellent), how would you rate your child’s global health status?

Please select one of the following proposals:

❑ 1

❑ 2

❑ 3

❑ 4

❑ 5

❑ 6

❑ 7

❑ 8

❑ 9

❑ 10

On a scale of 0 (very poor) to 10 (excellent), how would you rate your child's quality of life (daily activities, leisure, school integration, friends ...)?

Please select one of the following proposals:

- ❑ 1
- ❑ 2
- ❑ 3
- ❑ 4
- ❑ 5
- ❑ 6
- ❑ 7
- ❑ 8
- ❑ 9
- ❑ 10

Part 3

Perinatal Post-traumatic Stress Disorder Questionnaire (PPQ)

DeMier, R.L., Hynan, M.T., Harris, H.B., & Manniello, R.L. (1996). Perinatal stressors as predictors of symptoms of posttraumatic stress in mothers of infants at high- risk. *Journal of Perinatology,16,* 276- 280

Please circle « yes » if you have had any of these experiences since the birth of your baby. Circle "yes" only if the particular experience lasted for more than one month during this time.

1. Did you have several bad dreams of giving birth or of your baby's hospital stay?

Yes No

2. Did you have several upsetting memories of giving birth or of your baby's hospital stay?

Yes No

3. Did you have any sudden feelings as though your baby's birth was happening again?

Yes No

4. Did you try to avoid thinking about childbirth or your baby's hospital stay?

Yes No

5. Did you avoid doing things which might bring up feelings you had about childbirth or your baby's hospital stay (for example, not watching a TV show about babies)?

Yes No

6. Were you unable to remember parts of your baby's hospital stay?

Yes No

7. Did you lose interest in doing things you usually do? (For example, did you lose interest in your work or in your family?)

Yes No

8. Did you feel alone and removed from other people? (For example, did you feel like no one understood you?)

Yes No

9. Did it become more difficult for you to feel tenderness or love with others?

Yes No

10. Did you have unusual difficulty falling asleep or staying asleep?

Yes No

11. Were you more irritable or angry with others than usual?

Yes No

12. Did you have greater difficulties concentrating than before you gave birth?

Yes No

13. Did you feel more jumpy? (For example, did you feel more sensitive to noise, or more easily startled?)

Yes No

14. Did you feel more guilt about the childbirth than you felt you should have?

Yes No

<u>Part 4</u>

State – Trait Anxiety Inventory

Spielberger CD, Gorsuch RL, Lushene RE. 1970. STAI manual for the state-trait anxiety inventory. Palo Alto, CA: Consulting Psychologists Press.

Self-evaluation questionnaire STAI Form Y-1

A number of statements which people have used to describe themselves are given below. Read each statement and then circle the appropriate number below the statement to indicate how you feel *right now*, that is, *at this moment*. There are no right of wrong answers. Do not spend to much time on any one statement but give the answer which seems to describe your present feelings best.

I feel calm

1. Not at all 2. Somewhat 3. Moderately so 4. Very much so

I feel secure

1. Not at all 2. Somewhat 3. Moderately so 4. Very much so

I am tense

1. Not at all 2. Somewhat 3. Moderately so 4. Very much so

I feel strained

1. Not at all 2. Somewhat 3. Moderately so 4. Very much so

I feel at ease

1. Not at all 2. Somewhat 3. Moderately so 4. Very much so

I feel upset

1. Not at all 2. Somewhat 3. Moderately so 4. Very much so

I am presently worrying over possible misfortunes

1. Not at all 2. Somewhat 3. Moderately so 4. Very much so

I feel satisfied

1. Not at all 2. Somewhat 3. Moderately so 4. Very much so

I feel frightened

1. Not at all 2. Somewhat 3. Moderately so 4. Very much so

I feel comfortable

1. Not at all 2. Somewhat 3. Moderately so 4. Very much so

I feel self-confident

1. Not at all 2. Somewhat 3. Moderately so 4. Very much so

I feel nervous

1. Not at all 2. Somewhat 3. Moderately so 4. Very much so

I am jittery

1. Not at all 2. Somewhat 3. Moderately so 4. Very much so

I feel indecisive

1. Not at all 2. Somewhat 3. Moderately so 4. Very much so

I am relaxed

1. Not at all 2. Somewhat 3. Moderately so 4. Very much so

I feel content

1. Not at all 2. Somewhat 3. Moderately so 4. Very much so

I am worried

1. Not at all 2. Somewhat 3. Moderately so 4. Very much so

I feel confused

1. Not at all 2. Somewhat 3. Moderately so 4. Very much so

I feel steady

1. Not at all 2. Somewhat 3. Moderately so 4. Very much so

I feel pleasant

1. Not at all 2. Somewhat 3. Moderately so 4. Very much so

Self-evaluation questionnaire STAI Form Y-1

A number of statements which people have used to describe themselves are given below. Read each statement and then circle the appropriate number below of the statement to indicate how you *generally* feel. There are no right or wrong answers. Do not spend too much time on any one statement but give the answer which seems to describe how you *generally* feel.

I feel pleasant

1. Almost never 2. Sometimes 3. Often 4. Almost always

I feel nervous and restless

1. Almost never 2. Sometimes 3. Often 4. Almost always

I feel satisfied with myself

1. Almost never 2. Sometimes 3. Often 4. Almost always

I wish I could be as happy as others seem to be

1. Almost never 2. Sometimes 3. Often 4. Almost always

I feel like a failure

1. Almost never 2. Sometimes 3. Often 4. Almost always

I feel rested

1. Almost never 2. Sometimes 3. Often 4. Almost always

I am “calm, cool and collected”

1. Almost never 2. Sometimes 3. Often 4. Almost always

I feel that difficulties are piling up so that I cannot overcome them

1. Almost never 2. Sometimes 3. Often 4. Almost always

I worry too much over something that really doesn't matter

1. Almost never 2. Sometimes 3. Often 4. Almost always

I am happy

1. Almost never 2. Sometimes 3. Often 4. Almost always

I have disturbing thoughts

1. Almost never 2. Sometimes 3. Often 4. Almost always

I lack self-confidence

1. Almost never 2. Sometimes 3. Often 4. Almost always

I feel secure

1. Almost never 2. Sometimes 3. Often 4. Almost always

I make decisions easily

1. Almost never 2. Sometimes 3. Often 4. Almost always

I feel inadequate

1. Almost never 2. Sometimes 3. Often 4. Almost always

I am content

1. Almost never 2. Sometimes 3. Often 4. Almost always

Some unimportant thought runs through my mind and bothers me

1. Almost never 2. Sometimes 3. Often 4. Almost always

I take disappointments so keenly that I can't put them out of my mind

1. Almost never 2. Sometimes 3. Often 4. Almost always

I am a steady person

1. Almost never 2. Sometimes 3. Often 4. Almost always

I get in a state of tension or turmoil as I think over my recent concerns and interests

1. Almost never 2. Sometimes 3. Often 4. Almost always

Thank you for taking the time to complete this survey
